# A Countrywide Survey in Saudi Arabia Regarding the Knowledge and Attitude of Health Care Professionals about Coronavirus Disease (COVID-19)

**DOI:** 10.3390/ijerph17207415

**Published:** 2020-10-12

**Authors:** Abdulrahman A. Alduraywish, Kumar Chandan Srivastava, Deepti Shrivastava, Mohammed Ghazi Sghaireen, Abdalkarem F. Alsharari, Khalid Al-Johani, Mohammad Khursheed Alam

**Affiliations:** 1 Internal Medicine Department, Medical College, Jouf University, Sakaka 72345, Saudi Arabia; dr-aaad@ju.edu.sa; 2 Oral Medicine & Radiology, Department of Oral and Maxillofacial Surgery & Diagnostic Sciences, College of Dentistry, Jouf University, Sakaka 72345, Saudi Arabia; 3 Periodontics, Department of Preventive Dentistry, College of Dentistry, Jouf University, Sakaka 72345, Saudi Arabia; sdeepti20@gmail.com; 4 Prosthodontics, Prosthetic Dental Sciences, College of Dentistry, Jouf University, Sakaka 72345, Saudi Arabia; dr.mohammed.sghaireen@jodent.org; 5 Nursing Department, College of Applied Medical Sciences, Jouf University, Sakaka 72345, Saudi Arabia; afalsharari@ju.edu.sa; 6 Department of Oral Diagnostic Sciences, Faculty of Dentistry, King Abdulaziz University, Jeddah 80200, Saudi Arabia; kauoralmed@gmail.com; 7 Orthodontics, Department of Preventive Dentistry, College of Dentistry, Jouf University, Sakaka 72345, Saudi Arabia; dr.mohammad.alam@jodent.org

**Keywords:** COVID-19, knowledge, nomenclature, infection control, health care professionals, SARS-CoV2, coronavirus prevention, preparedness planning, 2019-nCoV

## Abstract

Coronavirus disease (COVID-19) has emerged as a pandemic. The updated knowledge and a positive attitude of health care professionals (HCPs) towards fighting any pandemic is the key to success. Thus, the present study aims to assess the knowledge and attitude of HCPs towards COVID-19 in the Kingdom of Saudi Arabia (KSA). A cross-sectional study was conducted across the KSA, covering its five geographical regions with a non-probability quota sample. Twenty-nine, close-ended questions evaluating the knowledge and attitude domain were included in the questionnaire. It was developed with the help of Qualtrics software and circulated among the HCPs through the electronic mode. We analyzed data from about 1040 HCPs using the statistical package of social sciences (SPSS) v.21. All variables were presented in number and percentages. Univariate and multivariate logistic regression was performed to explore the odds ratio (OR) and adjusted odds ratio (aOR) of independent variables for inadequate knowledge and attitude. Considering the “good” level of the respective domain, the HCPs have displayed better knowledge (48.2%) over attitude (33.8%). Female (aOR: 1.55; 95% CI: 1.15–2.09; *p* = 0.004), Diploma degree (aOR: 2.51; 95% CI: 1.64–3.83; *p* < 0.001), 7–10 years’ experience (aOR: 1.47; 95% CI: 1.01–2.15; *p* = 0.045) were at higher risk of having inadequate knowledge compared to their contemporaries. Among the sources, the Ministry of Health (MOH) website was the most popular source of information (76%). The knowledge and attitude of HCPs regarding COVID-19 was similar across all the regions of KSA. However, the continuing education program is warranted to fill the potential gap in knowledge for HCPs in higher-risk groups.

## 1. Introduction

With the accelerating stage of the pandemic coronavirus disease (COVID-19), the world has endorsed the slogan of “stay home–stay safe”. On the contrary, there are health care professionals (HCPs) who have opted to remain on the forefront to tackle the disease. The COVID-19 disease caused by severe acute respiratory syndrome coronavirus 2 (SARS-CoV-2), [1,2,3] has claimed the lives of many citizens worldwide. High human transmission of COVID-19 through contact or respiratory droplets has assisted in the rapid spread of the disease [3,4,5]. Other ways of transmission such as air, fecal, and vertical transmission have not yet been evidenced [6,7]. Since the virus remains alive on various inanimate objects from hour to days [8], it, hence, can be a potential source of cross-contamination.

The HCPs must be well updated with the current guidelines so they can treat the patient rationally. Meanwhile, they should also protect themselves from acquiring this disease. During a pandemic, the HCPs are over-burdened and are also in a stage of fear to acquiring the disease [9,10,11]. It becomes even more critical for any country to reconcile and boost them scientifically so they can defeat any disease by rational thinking and practical implication. It will not only help in flattening the curve of the pandemic, but also protect the HCPs from contacting the disease [10].

The world has seen many pandemics in the past and has overcome them. The key element is to follow the updated guidelines laid by various organizations such as the World Health Organization (WHO), the Center for Disease Control (CDC) and the Ministry of Health (MOH) of respective countries. The challenges faced by any nation are the proper dissemination of information so that not only HCPs, but also the community, should also strictly follow the policy and procedures [12]. The preparedness of the MOH to keep their HCPs updated with the recent advancement to deal with the diseases has a unique role to play.

In 2012, the Arabian Peninsula witnessed the Middle East respiratory syndrome-coronavirus (MERS-CoV). The Kingdom of Saudi Arabia (KSA) reported the maximum number of cases during that epidemic [13]. This MERS-CoV belongs to the same Coronavirus family, which has caused COVID-19 at present and SARS in the past [14]. Currently, in KSA there are 330,246 cases reported with 4512 deaths of COVID-19 [15]. At present, the need of the hour is for the HCPs to be provided with personal protective equipment (PPEs) and information required to treat the patients effectively. With the experience of disease transmission from patient to HCPs during MERS-CoV epidemic, the similar pattern is expected in COVID-19, and thus HCPs can act as a potential source of disease transmission [16,17]. Insufficient information and breach in the standard operating protocol (SOP) of infection control can be a source for disease transmission. In the past [16,17,18,19], cross-contamination from patients to HCPs and vice versa had occurred, and it is reported in the present time as well [20,21]. It would be a significant loss for any nation to lose HCPs who are the saviors. The shortage of HCPs will eventually worsen the condition. It is time to ponder how to protect the HCPs from acquiring this disease. Studies regarding knowledge and attitude of HCPs about COVID-19 have been done in other countries [22,23,24,25,26,27]. However, to the best of our knowledge, none has been done with Saudi Arabian HCPs. This study will help to assess the knowledge and attitude of HCPs towards COVID-19 and based on the evaluation; a reinforcement policy can be made for them to treat patients while protecting themselves effectively. Thus, the study aims to evaluate the knowledge and attitude of HCPs towards COVID-19 in KSA.

## 2. Materials and Methods

### 2.1. Study Identification

A cross-sectional study was planned with the prior approval from the institutional ethical board (14-07/41, dated 22 March 2020) and the data collection was carried out throughout the month of April 2020. The study was outlined and executed as per strengthening the reporting of observational studies in epidemiology (STROBE) guidelines [28].

### 2.2. Sample Identification

For this nationwide survey, the study population comprised a segment of health professionals including physicians, surgeons, nurses, pharmacists, physiotherapists, psychologists, and psychiatric practitioners in KSA. Other members of the HCPs team, including the dentist and dental auxiliaries, were excluded. Professionals comprising the allied health care team also remained in the exclusion criteria. We also omitted any other professionals who were not accredited to practice in KSA from the study.

At the time of the survey, due to the prevailing lockdown condition, a non-probability quota sample was drawn. To obtain a representative sample, due consideration was given to the characteristics such as geographic region, nature of the organization and type of work setup. During data analysis, additional factors such as age, gender, educational level, and work experience were considered. A total of 1236 HCPs had responded to the online questionnaire. The study excluded incomplete responses from 196 participants, leaving a sample of 1040, which was considered for analysis.

### 2.3. Questionnaire

As per protocol, firstly we identified the two domains about COVID-19 which we indented to assess among the HCPs. While reviewing the literature, we came across very few studies discussing this topic among dental professionals [29], residents of Hubei province of China [30], and health care workers (HCWs) [24]. Studies assessing the knowledge and attitude concerning MERS-CoV [31,32] and H1N1 [33] among HCWs also drew our attention. The questionnaire was adopted and later modified according to local requirements and variations. To accomplish this task, an expert committee was formed with members from the field of internal medicine, community medicine, a biostatistician, and bilingual translators. The information and guidelines issued from the global health agencies were taken as our source for framing the questions. With in-depth discussion sessions, the committee came up with the pre-final draft of the questionnaire, which was subjected to pilot testing. Taking into account the predominant Arabic speakers, translation (forward and backwards) of the questionnaire was done. To assess the internal consistency reliability of the scale, Cronbach’s alpha (α) of 0.81 was achieved, depicting an acceptable level of reliability and consistencies in response were assured. There were two sections in the questionnaire, with the first segment gathering sample characteristics. The second segment aims to assess the knowledge and attitude in two independent sub-sections consisting of 15 and 14 questions, respectively. All 29 questions were close-ended with two questions in each sub-section which were negatively worded.

### 2.4. Study Protocol

The primary channel for getting to the respondents was the questionnaire supported by Qualtrics software. A questionnaire’s link or Quick response (QR) code was shared via e-mail. The description of the study, including the purpose and objectives, were mentioned in the disclaimer of the questionnaire. The subjects were given the freedom to participate, and there was an assurance that it would not reveal their identity.

### 2.5. Data Management

The Knowledge segment comprised of 15 questions with three possible responses—“yes”, “no” and “I don’t know”. Initially, the responses were analyzed by calculating the percentages. Later, dummy codes of “1” or “0” were assigned for the right and wrong responses, respectively. Eventually, the total scores were calculated for individual respondent. Lastly, the class width is calculated by dividing the range (15 – 0 = 15) by 3 (desired number of the class interval) (Equation (1)). Based on the derived class width (5), the total score was converted into a three proportionate ordinal scale namely, poor (Score 0–5), moderate (score 6–10), and good (score 11–15). Equation (1):

(1) $$\mathrm{Class}\mathrm{width}(\mathrm{CW})=\frac{\mathrm{Maximum}\mathrm{Value}-\mathrm{Minimum}\mathrm{Value}}{\mathrm{Number}\mathrm{of}\mathrm{required}\mathrm{class}\mathrm{interval}}$$

The last segment of the questionnaire was dedicated to explore the attitude of HCPs towards various aspects of COVID-19 prevention, namely, infection control protocol, preventive and treatment measures. The attitude assessment section consisted of 14 questions, with two negatively worded questions in total. The response for each question was recorded on a 5-point Likert scale. In a positively worded question, a dummy code of “5” was assigned for “strongly agree” response, whereas “1” for assigned for a “strongly disagree” response. The response score was later added to derive the total attitude score. Similar to the knowledge domain, the attitude score was molded into an ordinal scale by applying Equation (1). Here the minimum and maximum attainable score by any respondent was 14 and 70, respectively. By dividing the range (70 – 14 = 56) by 3 (desired number of the class interval), a class width of 18 is derived. Using the class width, the total attitude score was converted into 3-point proportionate ordinal scale with categories namely, poor (score 14–32), moderate (score 33–51), and good (score 52–70).

### 2.6. Data Analysis

The data was analyzed using statistical package for the social sciences (SPSS) v.21 (IBM, Chicago, USA) All socio-demographic and professional characteristics of the participants were summarized as frequency and percentages. Univariate logistic regression was performed to assess the socio-demographic and professional factors associated with poor knowledge and attitude towards COVID-19. The factors which are significant at the univariate level were used for multivariate logistic regression.

## 3. Results

### 3.1. Descriptive Statistics of the Baseline Variables

The study evaluated the level of knowledge and attitude of 1040 HCPs employed in KSA. The maximum data pooled from the central region (28.6%) and least from the eastern region (5.2%) of KSA. The majority of the respondents belong to 31–40 years of age group (43.7%), and female predominance (67.4%) was recorded in the sample. A larger segment of subjects had a Bachelor’s Degree (46.5%) and professionals with >10 years of experience (38.5%) had maximum participation in the study. A vast majority of participants were working in the government sector (93.8%), predominately in the regional health center (48.7%). The MOH website was the most favored (76%) source of information followed by social media (50.2%) (Table 1). The responses of the participants for the knowledge and attitude domain about COVID-19 are expressed in Table 2 and Table 3, respectively. The distribution of responses among the independent variables is shown in Table S1.

### 3.2. Analysis about the Knowledge Domain

More than half (51.1%) of HCPs were found to have a “moderate” level of knowledge, and only 0.8% had “poor”. The respondents have shown exceptional knowledge regarding the incubation period (98.7%), symptoms (97.7%), identified respiratory droplets and contact as the mode of transmission (98.7%) and the current scenario about the vaccination (91.6%) of COVID-19. Worst response (9.8%) was observed when they were asked about the guidelines about hand hygiene, especially the duration aspect (Table 2).

The three-point knowledge scale was modified into a dichotomous variable. It was achieved by merging the “poor” and “average” scale into “inadequate” category, whereas “good” scale was renamed as “adequate” category. Later, the odds of having “inadequate knowledge” were assessed among the independent variables. On univariate logistic regression, demographic variables such as age, gender, nationality, and region were found to have a significant association with knowledge. Similarly, significant associations were observed with education and work-related parameters such as the type of work setup and work experience (Table 4). The youngest age group (20–30 years) had the highest risk of having inadequate knowledge (OR 2.73: 95% CI: 1.93–3.85) compared to the elder most age group (>40 years). Females had 88% of higher the probability of having inadequate knowledge compared to the male participant (OR 1.88: 95% CI: 1.44–2.44). Among the educational level, participants with a Bachelor’s Degree had the lowest risk for poor knowledge (OR 2.3: 95% CI: 1.65–3.22) compared to the other lower levels of education. In the current study, HCPs with experience of 1–3 years showed the lowest knowledge score (OR 2.05: 95% CI: 1.49–2.82) (Table 4).

All significant factors in univariate logistic regression were taken in multivariate logistic regression, and their relationship with knowledge levels was evaluated. Gender (aOR 1.55: 95% CI: 1.15–2.09), the educational level (aOR 2.51: 95% CI: 1.64–3.83), and work experience (aOR 1.47: 95% CI: 1.01–2.15) remained significant and their direction of the relationship also remain unchanged (Table 5).

### 3.3. Analysis about the Attitude Domain

A large number (63.8%) of HCPs had a moderate level of attitude; with the good and poor level recorded as 33.8% and 2.3%, respectively. On closer analysis of responses of individual questions, few observations are noteworthy. Respondents displayed a good attitude towards questions pertaining to PPEs (strongly agree—82%) and travel restrictions and large gatherings as precipitating factor (strongly agree—80.5%). On the contrary, poor attitude is shown for questions related to the usage of N95 mask (correct response—12.1%) and infection protocol to be followed during an epidemic or general situation (correct response—3.8%) (Table 3). For the statistical purpose, the “poor” and “moderate” level of attitude were merged and renamed as “inadequate”, whereas the “good” level was considered as “adequate”. In univariate logistic regression, the southern region had shown the least positive attitude (OR 1.49: 95% CI: 1–2.21), followed by northern region (OR 1.45: 95% CI: 1.03–2.04) (Table 4). As, no significant associations were found for the majority of the independent variable in univariate analysis, the multivariate analysis for attitude domain was not performed.

## 4. Discussion

COVID-19 has emerged as a pandemic with constantly changing concepts of the disease and its prevention. It is therefore considered necessary to keep the HCPs compliant with the recommendations issued by international and national health agencies. In the present study, the majority of the respondents demonstrated a moderate level of knowledge (51.1%) and attitude (63.8%) towards COVID-19. However, 48.2% and 33.8% of HCPs demonstrated a good level of knowledge and attitude, respectively. Studies with lower [34] as well as a higher [22] level of knowledge and attitude compared to the present study are reported. It can be contemplated that the higher levels of knowledge and attitude seen with the Chinese HCPs lies in the fact that the epicenter of COVID-19 was in China, and they had encountered this disease much earlier [22].

### 4.1. Information Source

An impressive fact has emerged from the present study, wherein the majority of the information (76%) regarding COVID-19 was imbibed by the HCPs from Saudi Arabia’s MOH website. It shows the alertness of the government to dig every possible area and update HCPs with the changing norms. It is meaningful to compare the present day scenario of moderate to good knowledge and attitude levels, with approximately 50% levels reported in a similar outbreak of MERS-CoV in 2012 [31]. This shows that the government acted promptly and judiciously by updating the policies and procedure for COVID-19 in the background of MERS-CoV. As the majority of the HCPs in the study were in their middle age, they were inclined towards evidence-based information (MOH website). Contrary to this, social media was a preferred source of information in another study [25]. This can be attributed to the fact that the majority of respondents were below 30 years of age (74.9%) with more dependence and ease with social media. Nevertheless, the usage of social media as the sole source, at times, can be misleading [34,35]; thus, multiple sources of information reduce the risk of inadequate knowledge as reported in the present study.

### 4.2. Assessment of the Knowledge Domain

The knowledge of HCPs about COVID-19′s nomenclature received a relatively lower response rate. The understanding and decoding of the virus were continuously updating. Hence, it could be a reasonable explanation for the observations made above. Initially, the microorganism was identified as 2019-nCoV, which was later renamed SARS-CoV-2 and assigned the COVID-19 terminology for the disease it caused [2,36]. The HCPs were well aware of the hallmark symptoms [37] and the mode of transmission [38] as the guideline laid by the MOH and WHO were incorporated as screening protocols in all health care centers [4,38,39,40,41]. Regarding treatment and vaccination, the HCPs were well updated about the fact that antibiotics are not the main line of treatment; instead, they are instituted as a part of palliative care. Presently, various therapeutics are under trial, but none of them has come with a promising result [4,42]. Similarly, the development of successful vaccination is under various phases of the trial [4,42]. The correct response rate (9.8%) regarding the duration of hand washing with the soap and water has raised grave concern. The WHO guideline instructs for 40–60 s of hand washing and permits a reduction to 20–40 s provided alcohol-based rub is used [38]. Where respondents were a giving high response rate for other sections, such response seems accidental. Confusion between the hand rub with hand wash and the consequence of a negatively worded question, might be the probable reason. Nonetheless, a more precise and positive reinforcement is warranted, as it is a key element of a preventive measure for hand hygiene.

In the present study, age has positively influenced the knowledge of HCPs about COVID-19. This is in agreement with Saqlain M et al. (2020) [25], where the middle and elderly age group were reported more knowledgeable compared to HCPs of younger age. Contrasting results were reported in a similar study done with HCPs in Uganda [26].

Interestingly, the level of education and years of experience have reported influencing the knowledge independently in an exponential manner. A convincing explanation of the phenomena mentioned above is the enhancement in the comprehension of the acquired knowledge seen with the advancing age. Moreover, a professional generally gain proficiency in the subject by attaining the highest level of qualification. In general, it has been observed that there is a sharpening of clinical knowledge over the increasing length of service. Collectively, such professionals tend to be more focused on evidence-based practice and keep updating themselves to acquire current knowledge [43]. However, other studies did not observe any significant association between knowledge with a level of education [26] and years of experience [25].

Additionally, gender has emerged as an independent risk factor for inadequate knowledge, wherein female HCPs have shown inadequate knowledge compared to their male counterpart. Conversely, female HCPs were reported more knowledgeable about MERS-CoV in KSA [31]. Interestingly no significant difference in the knowledge level among the various regions of KSA was reported. This re-emphasizes the positive role of government in disseminating the information about COVID-19 across the kingdom.

### 4.3. Assessment of the Attitude Domain

In the present study, we have found that the majority of the independent variables were non-influential on the level of attitude of HCPs. Similar observations were reported by Olum R et al. (2020) [26] and Saqlain et al. (2020) [25]. On the contrary, Zhang et al. (2020) [22] has found few influencing risk factors such as years of experience, overworked status, job category, and frontline status on attitude levels of HCPs. The timeframe (first week of February) and location (Henan—second in a row of the epicenter of COVID-19) of this study, made them include variables such as overworked status, job category, and frontline status, which were not included in the present study. As far as “years of experience” is concerned, it did not influence the attitude in our study, probably because, even the less experienced HCPs in KSA had witnessed the epidemic of MERS-CoV in 2012. Presumably, this ongoing risk of MERS-CoV has made almost all HCPs, irrespective of age, gender, educational level, or years of experience in KSA, display a positive attitude towards COVID-19 as well.

Reasonable knowledge is always displayed with a positive attitude [44]. In the present study, the HCPs were welcoming the professional education methods as well as keeping abreast of knowledge and disseminating the information. This is a positive attitude which helps to overcome any lacunae in an existing scenario. A key element for transmission or disease prevention is questioning the patient about his/her recent travel history, especially if visited countries facing an outbreak of disease. Not only international travel, even avoiding the large gathering in the local area is a preventive measure advised by the global (WHO and CDC) as well as regional (MOH; KSA) health agencies [45]. A larger portion of HCPs of the present study was shown to comply with these measures, and hence appeared in results to have a good attitude. Another fundamental concept of prevention is infection control measures, which are well established by health organizations [36,38,39]. Considering the contagious nature of the disease, the HCPs are equally susceptible to acquire the infection while treating patients. Thus, a good attitude towards strict compliance with the infection control protocol is necessary, and any negligence can put the HCPs into the risk of acquiring disease [23]. Cases of HCPs getting infected have been reported by various countries [46,47]. However, till date, no cases have been reported in KSA. Among the infection control measure, usage of PPE [38,48,49] is crucial, and the majority of the HCPs (82%) have responded well to wear PPE while dealing with the patients. N95 mask to be used by an undiagnosed patient can be an exaggeration of the present situation. The surgical mask instead of N95 can be provided to them to prevent dissemination of infection, which will prevent the shortage of the same for the HCPs [48]. The HCPs have responded incorrectly to a question where they showed agreement with the statement (negatively worded in the questionnaire) that “Standard precautions should be followed especially during the outbreak of the COVID-19”. HCPs should be reinforced to take universal and standard precautions at all time (CDC and WHO) irrespective of the disease outbreak [38]. This lies in the fact that COVID-19 is a novel disease, and currently, we are not aware of the possibility of transmission from an asymptomatic recovered patient as seen in the case of Hepatitis B, which has a clearly defined carrier state [50].

### 4.4. Strength, Limitations, and Future Directions of the Study

To the best of the available literature searches, the current study is the first of its kind to assess the knowledge and attitude of HCPs in KSA. Furthermore, this study is a nationwide survey as it has included the respondents from all five geographical regions of KSA. The observations in the study provide insight into the knowledge and attitude of HCPs during an early phase of the pandemic. This will facilitate the government to take proper actions and precautionary measures to limit its further spread. There are limitations in the study as well. Since this survey was conducted online, it has its disadvantage in terms of response bias. The current study opens the arena to conduct translation studies in other counties to have a global comparison.

## 5. Conclusions

From the present study, it is conclusive to say that the HCPs had adequate knowledge about COVID-19 in terms of the incubation period, symptoms, mode of transmission, and prevention of disease. HCPs have even shown a positive attitude towards the usage of PPE and travel restriction protocols. It is praiseworthy to note that the government has played a pivotal role in disseminating the information about COVID-19 to HCPs across Saudi Arabia. HCPs of >40 years of age, with more years of experience, and possessing an educational level of Master’s Degree and above are reported to have adequate knowledge. The current study proposes to have more continuing education program to overcome any knowledge gap of HCPs across KSA.

## Figures and Tables

**Table 1:** Descriptive Characteristics.

Variable	Responses		ƒ(n)(%)
**Sample Size (n)**			1040
Age	20–30 Years		371 (35.7)
	31–40 Years		454 (43.7)
	41–50 Years		151 (14.5)
	≥51 Years		64 (6.2)
Gender	Male		339 (32.6)
	Female		701 (67.4)
Nationality	Saudi		635 (61.1)
	Non Saudi		405 (38.9)
Region of Saudi Arabia	Central region (Riyadh and Qassim)		297 (28.6)
	Eastern region (Dammam, Jubail, Hassa, and others)		52 (5.2)
	Western region (Makkah, Jeddah, Taif, and Madinah)		224 (21.5)
	Northern region (Hail, Aljouf, Tabouk, and Arar)		291 (28)
	Southern region (Assir, Jazan, Najran, and Baha)		176 (16.9)
Educational level	Intern		71 (6.8)
	Diploma degree/Associate college		269 (25.9)
	Bachelor’s degree		484 (46.5)
	Master’s degree		138 (13.3)
	Doctorate/PhD		78 (7.5)
Work experience	1–3 Years		255 (24.5)
	4–6 Years		167 (16.1)
	7–10 Years		218 (21)
	˃10 Years		400 (38.5)
Nature of organization	Government		975 (93.8)
	Private		65 (6.3)
Type of work setup	Non-academic	Primary healthcare center	155 (14.9)
		Regional/Public hospital	506 (48.7)
		Specialized hospital/Referral center	197 (18.9)
		Military hospital/Medical clinics	45 (4.3)
		Private clinic	23 (2.2)
		Private hospital or Medical complex	35 (3.4)
	Academic	University hospital/Clinics	79 (7.6)
Source of information	Social media		522 (50.2)
	Professional colleague		191 (18.4)
	Ministry of health website		790 (76)
	Journals		201 (19.3)

Note: Responses are not mutually exclusive.

**Table 2:** Descriptive analysis of questions pertaining to the knowledge domain.

Category of Information	Question (Correct Response)	Response ƒ (%)		
		Yes	No	I Don’t Know
Nomenclature/Identification of causative organism	^‡^ Corona virus disease 2019 (COVID-19) is known as Severe Acute Respiratory Syndrome Coronavirus 2 (SARSCoV-2).	422 (40.6)	428 (41.2)	190 (18.3)
	Corona virus is the causative organism responsible for Middle East Respiratory Syndrome (MERS), Severe Acute Respiratory Syndrome (SARS) and COVID-19.	760 (73.1)	180 (17.3)	100 (9.6)
Origin of infection	In COVID-19, the Chinese horseshoe bats are considered as the most probable origin.	688 (66.2)	94 (9.0)	258 (24.8)
	^‡^ The main source of COVID-19 virus is plant.	13 (1.3)	895 (86.1)	132 (12.7)
	Does COVID-19 have any intermediate host?	420 (40.4)	289 (27.8)	331 (31.8)
Transmission	COVID-19 is transmitted by close contact with infected person or animal.	863 (83)	127 (12.2)	50 (4.8)
	COVID-19 can be transmitted from respiratory droplets and contact.	1026 (98.7)	14 (1.3)	0
Symptoms of infection	Incubation time for virus is 1–14 days.	1022 (98.3)	11 (1.1)	7 (0.7)
	Fever, dry cough, and shortness of breath are the hallmark symptoms of COVID-19.	1016 (97.7)	15 (1.4)	9 (0.9)
High-risk group	People with co-morbidity (Diabetes mellitus and other chronic diseases) are more likely to be infected with COVID-19.	894 (86)	86 (8.3)	60 (5.8)
Prognosis	COVID-19 has less fatality rate than Middle East respiratory syndrome related Coronavirus (MERS-CoV).	497 (47.8)	380 (36.5)	163 (15.7)
Investigation	Polymerase chain reaction (PCR) can be used to diagnose COVID-19.	605 (58.2)	82 (7.9)	353 (33.9)
Treatment	^‡^ Antibiotics are the first line treatment.	261 (25.1)	597 (57.4)	182 (17.5)
Prevention	^‡^ As per the guidelines issued from the health authorities, washing hands with soap and water for at least 30 s can help in the prevention of COVID-19.	924 (88.8)	102 (9.8)	14 (1.3)
	^‡^Vaccination of COVID-19 is available in the market.	14 (1.3)	953 (91.6)	73 (7)
Total Knowledge Score		Poor	Moderate	Good
		8 (0.8)	531 (51.1)	501 (48.2)

Note: ^‡^ Negatively worded questions.

**Table 3:** Descriptive analysis of questions pertaining to the attitude domain.

Category of Information	Question	Response n (%)				
		SD	D	n	A	SA
Awareness about COVID-19	Health-care professionals must update themselves with all the information about Corona virus disease 2019 (COVID-19).	39 (3.8)	8 (0.8)	21 (2)	225 (21.6)	747 (71.8)
	Any related information about COVID-19 should be disseminated among the peers and other health-care workers.	38 (3.7)	30 (2.9)	44 (4.2)	300 (28.8)	628 (60.4)
Precipitating factor	To comply with any local restrictions on travel, movement or large gatherings is one of the important ways of prevention from COVID-19.	27 (2.6)	4 (0.4)	14 (1.3)	158 (15.2)	837 (80.5)
Symptoms	People with fever, cough, and difficulty in breathing should seek medical attention.	26 (2.5)	3 (0/3)	17 (1.6)	258 (24.8)	736 (70.8)
Infection control protocol	Prevalence of COVID-19 can be reduced by active participation of health care workers in the hospital’s infection control program.	35 (3.4)	32 (3.1)	67 (6.4)	359 (34.5)	547 (52.6)
	Transmission of COVID-19 infection can be prevented using universal precautions given by the Center for Disease Control (CDC) and the World Health Organization (WHO).	30 (2.9)	12 (1.2)	37 (3.6)	343 (33)	618 (59.4)
	^‡^ Using N95 masks by the undiagnosed patients is critically important.	126 (12.1)	219 (21.1)	57 (15.1)	264 (25.4)	274 (26.3)
	Gowns, gloves, mask, and goggles must be used while dealing with COVID-19 patients.	26 (2.5)	8 (0.8)	10 (1)	143 (13.8)	853 (82)
	^‡^ Especially during the outbreak of COVID-19, every patient coming to the hospital, should be considered as infectious and all standard protocol should be adopted.	39 (3.8)	53 (5.1)	45 (4.3)	297 (28.6)	606 (58.3)
	Notify the receiving area about the patient’s diagnosis and necessary precautions should be taken as soon as possible before the patient’s arrival.	25 (2.4)	5 (0.5)	25 (2.4)	243 (23.4)	742 (71.3)
	Health care professionals who are transporting the patients should wear appropriate personal protective equipment and perform hand hygiene afterward.	26 (2.5)	6 (0.6)	8 (0.8)	151 (14.5)	849 (81.6)
Prevention	It is important to stay more than 1 m (3 feet) away from a person who is sick.	32 (3.1)	6 (0.6)	18 (1.7)	220 (21.2)	764 (73.5)
Treatment	Only suspected cases of COVID-19 patients should be kept in isolation.	69 (6.6)	81 (7.8)	40 (3.8)	309 (29.7)	541 (52)
	Intensive and emergency treatment should be given to COVID-19 diagnosed patients.	32 (3.1)	39 (3.8)	65 (6.3)	280 (26.9)	624 (60)
Total Attitude Score		Poor		Moderate		Good
		24 (2.3)		664 (63.8)		352 (33.8)

Note: SD—Strongly disagree, D—Disagree, N—Neutral, A—Agree, SA—Strongly Agree. **^‡^** Negatively worded question.

**Table 4:** Univariate Logistic regression analysis.

Parameters	Knowledge Domain				Attitude Domain			
	Adequate	Inadequate	Odds Ratio (CI)	*p* Value	Adequate	Inadequate	Odds Ratio (CI)	*p* Value
Age								
20–30 Yrs.	135 (36.4)	236 (63.6)	2.73 (1.93–3.85)	<0.001	124 (33.4)	247 (66.6)	1.13 (0.8–1.61)	0.483
31–40 Yrs.	235 (51.8)	219 (48.2)	1.45 (1.04–2.02)	0.026	150 (33)	304 (67)	1.15 (0.82–1.62)	0.409
>40 Yrs.	131 (60.9)	84 (39.1)	Reference		78 (36.3)	137 (63.7)	Reference	
Gender								
Male	199 (58.7)	140 (41.3)	Reference		122 (36)	217 (64)	Reference	
Female	302 (43.1)	399 (56.9)	1.88 (1.44–2.44)	<0.001	230 (32.8)	471 (67.2)	1.15 (0.88–1.51)	0.310
Educational Level								
Intern	26 (36.6)	45 (63.4)	3.61 (2.06–6.32)	<0.001	22 (31)	49 (69)	1.07 (0.6–1.9)	0.824
Diploma Degree/associate college	99 (36.8)	170 (63.2)	3.58 (2.46–5.22)	<0.001	89 (33.1)	180 (66.9)	0.97 (0.66–1.42)	0.874
Bachelor’s degree	230 (47.5)	254 (52.5)	2.3 (1.65–3.22)	<0.001	171 (35.3)	313 (64.7)	0.88 (0.62–1.23)	0.452
Above master’s degree	146 (67.6)	70 (32.4)	Reference		70 (32.4)	146 (67.6)	Reference	
Nationality								
Saudi	270 (42.5)	365 (57.5)	Reference		222 (35)	413 (65)	Reference	
Non-Saudi	231 (57)	174 (43)	0.56 (0.43–0.72)	<0.001	130 (32.1)	275 (67.9)	1.14 (0.87–1.48)	0.342
Region of Saudi Arabia								
Central	153 (51.5)	144 (48.5)	Reference		118 (39.7)	179 (60.3)	Reference	
Eastern	28 (53.8)	24 (46.2)	0.91 (0.5–1.64)	0.756	17 (32.7)	35 (67.3)	1.36 (0.73–2.53)	0.338
Western	105 (46.9)	119 (53.1)	1.2 (0.85–1.7)	0.294	72 (32.1)	152 (67.9)	1.39 (0.97–2)	0.075
Northern	146 (50.2)	145 (49.8)	1.06 (0.76–1.46)	0.745	91 (31.3)	200 (68.7)	1.45 (1.03–2.04)	**0.032**
Southern	69 (39.2)	107 (60.8)	1.65 (1.13–2.41)	0.010	54 (30.7)	122 (69.3)	1.49 (1–2.21)	**0.049**
Nature of organization								
Government	472 (48.4)	503 (51.6)	Reference		332 (34.1)	643 (65.9)	Reference	
Private	29 (44.6)	36 (55.4)	1.16 (0.7–1.93)	0.554	20 (30.8)	45 (69.2)	1.16 (0.67–2)	0.588
Type of work setup								
Health care centre	313 (45.8)	371 (54.2)	1.33 (1.03–1.71)	0.031	245 (35.8)	439 (64.2)	0.77 (0.58–1.01)	0.063
Multi-Speciality hospital	188 (52.8)	168 (47.2)	Reference		107 (30.1)	249 (69.9)	Reference	
Work experience								
1–3 Years	102 (40)	153 (60)	2.05 (1.49–2.82)	<0.001	79 (31)	176 (69)	1.28 (0.92–1.79)	0.147
4–6 Years	75 (44.9)	92 (55.1)	1.68 (1.17–2.41)	0.005	48 (28.7)	119 (71.3)	1.43 (0.96–2.11)	0.077
7–10 Years	93 (42.7)	125 (57.3)	1.84 (1.32–2.57)	<0.001	79 (36.2)	139 (63.8)	1.01 (0.72–1.43)	0.949
>10 Years	231 (57.8)	169 (42.3)	Reference		146 (36.5)	254 (63.5)	Reference	
Number of sources of information								
1	269 (45.1)	327 (54.9)	1.71 (1.2–2.44)	0.003	201 (33.7)	395 (66.3)	0.98 (0.68–1.42)	0.926
2	139 (48.8)	146 (51.2)	1.48 (1–2.19)	0.050	98 (34.4)	187 (65.6)	0.95 (0.63–1.44)	0.822
>2	93 (58.5)	66 (41.5)	Reference		53 (33.3)	106 (66.7)	Reference	

Note: CI—Confidence interval; Results are expressed in Number (%).

**Table 5:** Multivariate Logistic regression analysis.

Parameters	Knowledge Domain	
	aOR (CI)	*p* Value
Age		
20–30 Yrs.	1.44 (0.83–2.48)	0.195
31–40 Yrs.	1.02 (0.7–1.51)	0.902
>40 Yrs.	Reference	
Gender		
Male	Reference	
Female	1.55 (1.15–2.09)	0.004
Educational level		
Intern	1.56 (0.8–3.03)	0.195
Diploma degree/Associate college	2.51 (1.64–3.83)	<0.001
Bachelor’s degree	1.35 (0.9–2.01)	0.145
Above Master’s degree	Reference	
Nationality		
Saudi	Reference	
Non-Saudi	0.76 (0.55–1.05)	0.095
Region of Saudi Arabia		
Central region	Reference	
Eastern region	0.84 (0.45–1.56)	0.576
Western region	1.02 (0.7–1.49)	0.908
Northern region	1.14 (0.8–1.62)	0.462
Southern region	1.41 (0.94–2.1)	0.094
Type of work setup		
Health care centre	1.13 (0.85–1.51)	0.405
Multi-Speciality hospital	Reference	
Work experience		
1–3 Years	1.48 (0.91–2.42)	0.115
4–6 Years	1.52 (0.97–2.37)	0.067
7–10 Years	1.47 (1.01–2.15)	0.045
˃10 Years	Reference	
Number of Sources for information		
1	1.19 (0.81–1.75)	0.379
2	1.14 (0.75–1.74)	0.539
>2	Reference	

Note: CI—Confidence interval; aOR—adjusted Odds ratio.

## Data Availability

The data set used in the current study will be made available on request from (Dr. Kumar Chandan Srivastava; drkcs.omr@gmail.com.

## Supplementary Materials

The following are available online at https://www.mdpi.com/1660-4601/17/20/7415/s1, Table S1: Frequency distribution of knowledge and attitude scores among the independent variables.

## References

[1] Wu D., Wu T., Liu Q., Yang Z. (2020). The SARS-CoV-2 outbreak: What we know. Int. J. Infect. Dis..

[2] Guo Y.-R., Cao Q.-D., Hong Z.-S., Tan Y.-Y., Chen S.-D., Jin H.-J., Tan K.-S., Wang D.Y., Yan Y. (2020). The origin, transmission and clinical therapies on coronavirus disease 2019 (COVID-19) outbreak–an update on the status. Mil. Med Res..

[3] Sohrabi C., Alsafi Z., O’Neill N., Khan M., Kerwan A., Al-Jabir A., Iosifidis C., Agha R. (2020). World Health Organization declares global emergency: A review of the 2019 novel coronavirus (COVID-19). Int. J. Surg..

[4] World Health Organization Modes of Transmission of Virus Causing COVID-19: Implications for IPC Precaution Recommendations: Scientific Brief. https://www.who.int/news-room/commentaries/detail/modes-of-transmission-of-virus-causing-covid-19-implications-for-ipc-precaution-recommendations.

[5] Shereen M.A., Khan S., Kazmi A., Bashir N., Siddique R. (2020). COVID-19 infection: Origin, transmission, and characteristics of human coronaviruses. J. Adv. Res..

[6] Karimi-Zarchi M., Neamatzadeh H., Dastgheib S.A., Abbasi H., Mirjalili S.R., Behforouz A., Ferdosian F., Bahrami R. (2020). Vertical Transmission of Coronavirus Disease 19 (COVID-19) from Infected Pregnant Mothers to Neonates: A Review. Fetal Pediatr. Pathol..

[7] Peng X., Xu X., Li Y., Cheng L., Zhou X., Ren B. (2020). Transmission routes of 2019-nCoV and controls in dental practice. Int. J. Oral Sci..

[8] Van Doremalen N., Bushmaker T., Morris D.H., Holbrook M.G., Gamble A., Williamson B.N., Tamin A., Harcourt J.L., Thornburg N.J., Gerber S.I. (2020). Aerosol and Surface Stability of SARS-CoV-2 as Compared with SARS-CoV-1. New Engl. J. Med..

[9] Tan B.Y., Chew N.W., Lee G.K., Jing M., Goh Y., Yeo L.L., Zhang K., Chin H.-K., Ahmad A., Khan F.A. (2020). Psychological Impact of the COVID-19 Pandemic on Health Care Workers in Singapore. Ann. Intern. Med..

[10] Adams J.G., Walls R.M. (2020). Supporting the Health Care Workforce During the COVID-19 Global Epidemic. JAMA.

[11] Nemati M., Ebrahimi B., Nemati F. (2020). Assessment of Iranian Nurses’ Knowledge and Anxiety Toward COVID-19 During the Current Outbreak in Iran. Arch. Clin. Infect. Dis..

[12] Al-Hanawi M.K., Angawi K., Alshareef N., Qattan A.M.N., Helmy H.Z., Abudawood Y., AlQurashi M., Kattan W.M., Kadasah N.A., Chirwa G.C. (2020). Knowledge, Attitude and Practice Toward COVID-19 Among the Public in the Kingdom of Saudi Arabia: A Cross-Sectional Study. Front. Public Health.

[13] Aly M., Elrobh M., Alzayer M., Aljuhani S., Balkhy H. (2017). Occurrence of the Middle East Respiratory Syndrome Coronavirus (MERS-CoV) across the Gulf Corporation Council countries: Four years update. PLoS ONE.

[14] Barry M., Al Amri M., A Memish Z. (2020). COVID-19 in the Shadows of MERS-CoV in the Kingdom of Saudi Arabia. J. Epidemiol. Glob. Health.

[15] World Health Organization Coronavirus Disease 2019 (COVID-19): Situation Report, 90. https://www.who.int/emergencies/diseases/novel-coronavirus-2019/situation-reports.

[16] Elkholy A.A., Grant R., Assiri A.M., Elhakim M., Malik M.R., Van Kerkhove M.D. (2020). MERS-CoV infection among healthcare workers and risk factors for death: Retrospective analysis of all laboratory-confirmed cases reported to WHO from 2012 to 2 June 2018. J. Infect. Public Health.

[17] Bhat M., Ghali P., Deschenes M., Wong P. (2012). Hepatitis B and the Infected Health Care Worker: Public Safety at What Cost?. Can. J. Gastroenterol..

[18] Hunter J.C., Nguyen D., Aden B., Al Bandar Z., Al Dhaheri W., Abu Elkheir K., Khudair A., Al Mulla M., El Saleh F., Imambaccus H. (2016). Transmission of Middle East Respiratory Syndrome Coronavirus Infections in Healthcare Settings, Abu Dhabi. Emerg. Infect. Dis..

[19] Weinstein R.A., Bridges C.B., Kuehnert M.J., Hall C.B. (2003). Transmission of Influenza: Implications for Control in Health Care Settings. Clin. Infect. Dis..

[20] Wang J., Zhou M., Liu F. (2020). Reasons for healthcare workers becoming infected with novel coronavirus disease 2019 (COVID-19) in China. J. Hosp. Infect..

[21] Schwartz J., King C.-C., Yen M.-Y. (2020). Protecting Healthcare Workers During the Coronavirus Disease 2019 (COVID-19) Outbreak: Lessons From Taiwan’s Severe Acute Respiratory Syndrome Response. Clin. Infect. Dis..

[22] Zhang M., Zhou M., Tang F., Wang Y., Nie H., Zhang L., You G. (2020). Knowledge, attitude, and practice regarding COVID-19 among healthcare workers in Henan, China. J. Hosp. Infect..

[23] Giao H., Han N.T., Van Khanh T., Ngan V.K., Van Tam V., An P.L. (2020). Knowledge and attitude toward COVID-19 among healthcare workers at District 2 Hospital, Ho Chi Minh City. Asian Pac. J. Trop. Med..

[24] Modi P.D., Nair G., Uppe A., Modi J., Tuppekar B., Gharpure A.S., Langade D. (2020). COVID-19 Awareness Among Healthcare Students and Professionals in Mumbai Metropolitan Region: A Questionnaire-Based Survey. Cureus.

[25] Saqlain M., Munir M.M., Rehman S.U., Gulzar A., Naz S., Ahmed Z., Tahir A.H., Mashhood M. (2020). Knowledge, attitude, practice and perceived barriers among healthcare workers regarding COVID-19: A cross-sectional survey from Pakistan. J. Hosp. Infect..

[26] Olum R., Chekwech G., Wekha G., Nassozi D.R., Bongomin F. (2020). Coronavirus Disease-2019: Knowledge, Attitude, and Practices of Health Care Workers at Makerere University Teaching Hospitals, Uganda. Front. Public Health.

[27] Nepal R., Sapkota K., Adhikari K., Paudel P., Adhikari B., Paudyal N., Sapkota K., Nepal R. (2020). Knowledge, attitude and practice regarding COVID-19 among healthcare workers in Chitwan, Nepal. J. Hosp. Infect..

[28] Von Elm E., Altman D.G., Egger M., Pocock S.J., Gøtzsche P.C., Vandenbroucke J.P. (2007). The Strengthening the Reporting of Observational Studies in Epidemiology (STROBE) Statement. Epidemiology.

[29] Khader Y., Al Nsour M., Al-Batayneh O.B., Saadeh R., Abbas H.B., Alfaqih M., Al-Azzam S., Alshurman B.A. (2020). Dentists’ Awareness, Perception, and Attitude Regarding COVID-19 and Infection Control: Cross-Sectional Study Among Jordanian Dentists. JMIR Public Health Surveill..

[30] Zhong B.-L., Luo W., Li H.-M., Zhang Q.-Q., Liu X.-G., Li W.-T., Li Y. (2020). Knowledge, attitudes, and practices towards COVID-19 among Chinese residents during the rapid rise period of the COVID-19 outbreak: A quick online cross-sectional survey. Int. J. Biol. Sci..

[31] Asaad A.M., El-Sokkary R.H., Alzamanan M.A., El-Shafei M. (2020). Knowledge and attitudes towards Middle East respiratory syndrome-coronavirus (MERS-CoV) among health care workers in south-western Saudi Arabia. East Mediterr. Health J..

[32] Baseer M.A., Althomairy S.A., Assery M., AlSaffan A.D. (2018). Knowledge and attitude of dental health professionals about middle east respiratory syndrome in Saudi Arabia. J. Int. Soc. Prev. Commun. Dent..

[33] Askarian M., Danaei M., Vakili V. (2013). Knowledge, Attitudes, and Practices Regarding Pandemic H1N1 Influenza Among Medical and Dental Residents and Fellowships in Shiraz, Iran. Int. J. Prev. Med..

[34] Bhagavathula A.S., Aldhaleei W.A., Rahmani J., Mahabadi M.A., Bandari D.K. (2020). Novel Coronavirus (COVID-19) Knowledge and Perceptions: A Survey on Healthcare workers. medRxiv.

[35] Srivastava K.C., Shrivastava D., Chhabra K.G., Naqvi W., Sahu A. (2020). Facade of media and social media during COVID-19: A review. Int. J. Res. Pharm. Sci..

[36] ArrayExpress—A Database of Functional Genomics Experiments. http://www.ebi.ac.uk/arrayexpress/.

[37] Gostic K.M., Gomez A.C.R., O Mummah R., Kucharski A.J., O Lloyd-Smith J. (2020). Estimated effectiveness of symptom and risk screening to prevent the spread of COVID-19. eLife.

[38] World Health Organization (2020). Infection Prevention and Control During Health Care When Novel Coronavirus (nCoV) Infection is Suspected Interim Guidance.

[39] World Health Organization (2020). Coronavirus Disease (COVID-19) Outbreak: Rights, Roles and Responsibilities of Health Workers, Including Key Considerations for Occupational Safety and Health.

[40] Novel Corona Virus (2019-nCoV) Infection Guidelines. https://www.moh.gov.sa/en/CCC/healthp/regulations/Documents/Novel%20Corona%20Virus%20Infection%20Guidelines.pdf.

[41] Coronavirus Disease 19 (COVID-19) Guidelines. https://www.moh.gov.sa/CCC/healthp/regulations/Documents/Coronavirus%20Disease%202019%20Guidelines%20v1.1..pdf.

[42] Xiao Y., Torok M.E. (2020). Taking the right measures to control COVID-19. Lancet Infect. Dis..

[43] Boström A.-M., Sommerfeld D.K., Stenhols A.W., Kiessling A. (2018). Capability beliefs on, and use of evidence-based practice among four health professional and student groups in geriatric care: A cross sectional study. PLoS ONE.

[44] McEachan R., Taylor N., Harrison R., Lawton R., Gardner P., Conner M. (2016). Meta-Analysis of the Reasoned Action Approach (RAA) to Understanding Health Behaviors. Ann. Behav. Med..

[45] World Health Organization (2020). Key Considerations for Repatriation and Quarantine of Travellers in Relation to the Outbreak of Novel Coronavirus 2019-nCoV.

[46] Lancet T. (2020). COVID-19: Protecting health-care workers. Lancet.

[47] Ran L., Chen X., Wang Y., Wu W., Zhang L., Tan X. (2020). Risk Factors of Healthcare Workers with Corona Virus Disease 2019: A Retrospective Cohort Study in a Designated Hospital of Wuhan in China. Clin. Infect. Dis..

[48] World Health Organization (2020). Rational Use of Personal Protective Equipment for Coronavirus Disease (COVID-19) and Considerations during Severe Shortages: Interim Guidance, 6 April 2020.

[49] World Health Organization Report of the WHO-China Joint Mission on Coronavirus Disease 2019 (Covid-19). https://www.who.int/publications-detail/report-of-the-who-china-joint-mission-on-coronavirus-disease-2019-(covid-19).

[50] Sharma S., Saini N., Chwla Y. (2005). Hepatitis B Virus: Inactive carriers. Virol. J..

