# Supplementary material for: A Countrywide Survey in Saudi Arabia Regarding the Knowledge and Attitude of Health Care Professionals about Coronavirus Disease (COVID-19)
Source: Int J Environ Res Public Health. 2020 Oct 12;17(20):7415. doi: 10.3390/ijerph17207415 (PMC7600160; doi:10.3390/ijerph17207415)
Supplement: Supplementary file 1 [file ijerph-17-07415-s001.pdf]

Supplementary Table: Frequency distribution of knowledge and attitude scores among the independent variables.

Variable	Knowledge			Attitude		
	Poor	Moderate	Good	Poor	Moderate	Good
Age						
20–30 Years	4 (1.1)	232 (62.5)	135 (36.4)	6 (1.6)	241 (65)	124 (33.4)
31–40 Years	4 (0.9)	215 (47.4)	235 (51.8)	13 (2.9)	291 (64.1)	150 (33)
41–50 Years	0 (0)	66 (43.7)	85 (56.3)	3 (2)	97 (64.2)	51 (33.8)
≥ 51 Years	0 (0)	18 (28.1)	46 (71.9)	2 (3.1)	35 (54.7)	27 (42.2)
Gender						
Male	3 (0.9)	137 (40.4)	199 (58.7)	14 (4.1)	203 (59.9)	122 (36)
Female	5 (0.7)	394 (56.2)	302 (43.1)	10 (1.4)	461 (65.8)	230 (32.8)
Nationality						
Saudi	7 (1.1)	358 (56.4)	270 (42.5)	7 (1.1)	406 (63.9)	222 (35)
Non-Saudi	1 (0.2)	173 (42.7)	231 (57)	17 (4.2)	258 (63.7)	130 (32.1)
Region of Saudi Arabia						
Central	3 (1)	141 (47.5)	153 (51.5)	11 (3.7)	168 (56.6)	118 (39.7)
Eastern	0 (0)	24 (46.2)	28 (53.8)	0 (0)	35 (67.3)	17 (32.7)
Western	1 (0.4)	118 (52.7)	105 (46.9)	3 (1.3)	149 (66.5)	72 (32.1)
Northern	3 (1)	142 (48.8)	146 (50.2)	8 (2.7)	192 (66)	91 (31.3)
Southern	1 (0.6)	106 (60.2)	69 (39.2)	2 (1.1)	120 (68.2)	54 (30.7)
Educational Level						
Intern	2 (2.8)	43 (60.6)	26 (36.6)	2 (2.8)	47 (66.2)	22 (31)
Diploma / Associate College	5 (1.9)	165 (61.3)	99 (36.8)	1 (0.4)	179 (66.5)	89 (33.1)
Bachelor's degree	1 (0.2)	253 (52.3)	230 (47.5)	12 (2.5)	301 (62.2)	171 (35.3)
Master's Degree	0 (0)	57 (41.3)	81 (58.7)	5 (3.6)	95 (68.8)	38 (27.5)
Doctorate / PhD	0 (0)	13 (16.7)	65 (83.3)	4 (5.1)	42 (53.8)	32 (41)
Work Experience						
1–3 Years	2 (0.8)	151 (59.2)	102 (40)	2 (0.8)	174 (68.2)	79 (31)
4–6 Years	1 (0.6)	91 (54.5)	75 (44.9)	8 (4.8)	111 (66.5)	48 (28.7)
7–10 Years	3 (1.4)	122 (56)	93 (42.7)	4 (1.8)	135 (61.9)	79 (36.2)
> 10 Years	2 (0.5)	167 (41.8)	231 (57.8)	10 (2.5)	244 (61)	146 (36.5)
Nature of Organization						
Government	8 (0.8)	495 (50.8)	472 (48.4)	20 (2.1)	623 (63.9)	332 (34.1)
Private	0 (0)	36 (55.4)	29 (44.6)	4 (6.2)	41 (63.1)	20 (30.8)
Type of Work Setup						
Primary Health Centre	2 (1.3)	80 (51.6)	73 (47.1)	3 (1.9)	104 (67.1)	48 (31)
Regional Hospital	5 (1)	272 (53.8)	229 (45.3)	6 (1.2)	312 (61.7)	188 (37.2)
Specialized Hospital	1 (0.5)	100 (50.8)	96 (48.7)	4 (2)	141 (71.6)	52 (26.4)
Military Hospital	0 (0)	19 (42.2)	26 (57.8)	0 (0)	26 (57.8)	19 (42.2)
Private Clinic	0 (0)	12 (52.2)	11 (47.8)	1 (4.3)	13 (56.5)	9 (39.1)
Private Hospital	0 (0)	17 (48.6)	18 (51.4)	3 (8.6)	21 (60)	11 (31.4)
University health centre	0 (0)	31 (39.2)	48 (60.8)	7 (8.9)	47 (59.5)	25 (31.6)
Number of Source of Information						
1	5 (0.8)	322 (54)	269 (45.1)	11 (1.8)	384 (64.4)	201 (33.7)
2	3 (1)	143 (50.1)	139 (48.7)	7 (2.4)	180 (63.1)	98 (34.3)
>2	0 (0)	66 (41.5)	93 (58.5)	6 (3.7)	100 (62.8)	53 (33.3)
